# Expression and distribution patterns of VEGF, TGF‐β_1_ and HIF‐1α in the ovarian follicles of Tibetan sheep

**DOI:** 10.1002/vms3.907

**Published:** 2022-08-31

**Authors:** Yajun Guo, Miaomiao Liu, Jos Dorian Lawson Mfoundou, Xinrong Wang

**Affiliations:** ^1^ College of Animal Science and Technology Gansu Agricultural University Lanzhou China; ^2^ National Engineering Laboratory for Animal Breeding, Key Laboratory of Animal Genetics and Breeding of the Ministry of Agriculture College of Animal Science and Technology China Agricultural University Beijing China

**Keywords:** follicles, granulosa cells, HIF‐1α, TGF‐β_1_, Tibetan sheep, VEGF

## Abstract

**Background:**

Hypoxia‐inducible factor‐1α (HIF‐1α), vascular endothelial growth factor (VEGF) and transforming growth factor β_1_ (TGF‐β_1_) are multifunctional growth factors that play an important role in follicular growth and development. However, its biological function in the follicular development of Tibetan sheep at different stages has not been described.

**Objectives:**

The purpose of this study was to investigate the effect of VEGF, TGF‐β_1_ and HIF‐1α expression and distribution on the development of follicles of different sizes.

**Methods:**

Immunohistochemistry (IHC), western blot (WB) and quantification real‐time polymerase chain reaction (qRT‐PCR) were used to detect the localisation and quantitative expression of VEGF, TGF‐β_1_ and HIF‐1α proteins and mRNA in small‐ (< 3 mm), medium‐ (3 mm < diameter < 5 mm)‐, and large‐ (> 5 mm) sized follicles.

**Results:**

The results showed that the proteins VEGF, TGF‐β_1_ and HIF‐1α, as well as their mRNA, were expressed in follicles. However, the expression in medium‐sized follicles was significantly higher than that in large‐ and small‐sized follicles (*p* <0.05). IHC also showed that the proteins VEGF, TGF‐β_1_, and HIF‐1α were distributed in granulosa cells (GCs) in small‐, medium‐, and large‐sized follicles.

**Conclusions:**

This study indicates that VEGF, TGF‐β_1_ and HIF‐1α, which operate in an autocrine or paracrine manner with the GCs, influence the follicular progressive growth, suggesting that these growth factors are closely associated with the follicular growth and development in ovarian.

## Graphical abstract

The distribution and expression of TGF‐β_1_ in different follicle sizes suggest that it is related to follicle growth and development.

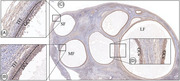

## 1 BACKGROUND

Tibetan sheep may be found mostly in the plateau pastoral region, which is 3000 m above sea level. The environmental adaptation of animals living has been linked to high altitude and hypoxia with the most significant effect on fertility (Lim et al., 2021; Nishimura & Okuda, 2015). Follicular development is a dynamic and complex process. The physiological process of follicular growth and maturation is precise and subtle. Meanwhile, some studies have found that when the follicles’ diameter reaches 3 mm, their growth periods are launched, and the dominating follicles develop from around 8 mm to preovulatory follicles, with alterations in cumulus‐oocyte complex (COC) protein production (Dieleman et al., 2002). GCs begin as a layer of follicle cells surrounding immature oocytes and continue to proliferate and differentiate until ovulation (Ernst et al., 2017). Furthermore, the proliferation of GCs is required not only for follicular growth but also for the creation of a unique microenvironment for oocyte maturation (Gougeon, 2010). During the growth of GCs, the follicles become sensitive to variations in follicle stimulating hormone (FSH) cycles during GCs proliferation (Henríquez et al., 2017). In response to a gonadotropin surge during ovulation, GCs may generate the VEGF (Stouffer et al., 2001). Simultaneously, HIF‐1α and VEGF are essential for follicular development and are crucial inducers of angiogenesis (Lim et al., 2021). In mammals, known cellular signalling pathways such as PI3K/AKT, AKT/mTOR, TGF‐β and insulin growth factor (IGF) signalling regulated follicular development (Ernst et al., 2017). Another study has reported that there was HIF‐1α expression in the ovaries, indicating that HIF‐1α is involved in physiological function regulation in Yaks (Fan et al., 2020). TGF‐β_1_ is an important growth factor that affects ovarian functions such as follicular development, steroidogenesis, ovulation, luteinisation and female fertility, among others (Knight & Glister, 2006). So far, VEGF, TGF‐β_1_ and HIF‐1α have not been utilised to comprehensively define the various sizes of follicles, nor have the influences of three factors on ovarian follicles been thoroughly investigated in Tibetan sheep. To evaluate the potential contribution of VEGF, TGF‐β_1_ and HIF‐1α in regulating follicular development under hypoxic environments in Tibetan sheep, we investigated their expression characteristics and immunolocalisation patterns during different follicular sizes. This research indicates that VEGF, TGF‐β_1_ and HIF‐1α are involved in GCs proliferation and differentiation to stimulate follicular growth and development, providing a reference for follicular development, ovarian function and pathologies.

## 2 MATERIALS AND METHODS

### 2.1 Ethics statement

All animal experiments were conducted according to the guidelines for the care and use of experimental animals established by the Ministry of Science and Technology of the People's Republic of China (approval number: 2006–398), and the work was approved by the Experimental Animal Care and Use Committee of the Gansu Agricultural University (GAU‐LL‐2020‐48), Lanzhou, China.

### 2.2 Experimental animals and sample collection

In the experiment, many ovaries were obtained from a local slaughterhouse (Xining, China). Twenty‐four healthy purebred Tibetan ewes were selected; their ovarian samples were collected (bilateral ovary). Next, the follicles obtained from these ovaries were grouped according to size into small‐ (diameter < 3 mm), medium‐ (3< diameter < 5 mm) and large‐sized (diameter > 5 mm) follicles (Jing et al., 2017). Some samples (follicles of different sizes) were frozen in liquid nitrogen to extract RNA and proteins, the other samples (ovarian tissue) were fixed in 4% paraformaldehyde for approximately 48 h and then embedded in paraffin.

### 2.3 RNA extraction and reverse transcription

TRIzol reagent (TransGen, Beijing, China) was used to extract total RNA from the different follicles of Tibetan sheep. A NanoDrop spectrophotometer (Thermo Fisher Scientific, Wilmington, DA, USA) was used to detect the concentration and quality of the extracted total RNA. Next, the RNA samples were reverse transcribed into cDNA using a reverse cDNA transcription kit (TransGen Biotech Co., Ltd., Beijing, China) according to the manufacturer's instructions.

### 2.4 qRT‐PCR assay

According to the *HIF‐1α*, *VEGF* and *TGF‐β_1_* gene sequences of sheep (*Ovis aries*) published in GenBank, primers were designed using NCBI Primer‐BLAST. Beta‐actin (β‐actin) was used as the internal reference gene, and all primers used were sent to the Wuhan Servicebio Technology Co., Ltd (Wuhan, China) for synthesis (the specific primer sequence information is shown in Table 1). The reaction system of 25 μl includes a 2 × qPCR Mix of 12.5 μl, the gene‐specific primer of 1.0 μl, reverse transcription to produce cDNA of 2.5 μl and ddH_2_O of 8.0 μl. The reaction conditions were as follows: pre‐denaturation (95°C, 2 min), denaturation (95°C, 15 s), annealing (60°C, 15 s), extension (65°C, 15 s), cycle (40 times), 65°C–95°C preparation melting curve and the mRNA expression of *VEGF*, *TGF‐β_1_* and *HIF‐1α* were calculated using the 2^−ΔΔCt^ method (Livak & Schmittgen, 2001).

**TABLE 1:** List of the primers used in qRT‐PCR

Gene	Accession no	Primer sequence (5′‐3′)	Product length (bp)
*VEGF*	NM_001025110.1	F: CATTGGAGCCTTGCCTTGC R: AGAAGCTGCGCTGGTAGACAT	127
*HIF‐1α*	XM_027971915.1	F: CGACCCTGCACTCAACCAAG R: CTGTTAGGCTCAGGTGAACTTTGT	147
*TGF‐β_1_*	NM_001009400.2	F: CAATTCCTGGCGCTACCTCA R: GAACTGAACCCGTTGATGTCC	195
*β‐actin*	NM_001009784.3	F: GCAGATGTGGATCAGCAAGC R: TCTCGTTTTCTGCGCAAGTT	133

### 2.5 Western blot analysis

RIPA buffer (Solarbio, Beijing, China) was used for extraction. A bicinchoninic acid (BCA) detection kit was used to detect the protein extract concentration. An equal amount of protein lysate was mixed with 5× sodium dodecyl sulphate (SDS) buffer and boiled for 10 min to denature. The denatured protein was separated using SDS‐PAGE gels. VEGF, TGF‐β_1_, HIF‐1α antibodies (Bioss, Beijing, China; bs‐1665R, bs‐0086R, bs‐0734R) and anti‐β‐actin antibody were incubated with an IgG antibody and heated at 37°C for 2 h. Finally, the intensity of each protein band was measured using AlphaEaseFC image analysis software (Alpha Innotech, CA, USA). The detailed experimental procedures for western blotting have been described previously (Li et al., 2020).

### 2.6 IHC staining

Paraffin‐embedded follicle sections were deparaffinised and dehydrated; they were washed with phosphate‐buffer saline (PBS), antigen was repaired in a microwave, and blocked with 5% BSA for 30 min at room temperature. The VEGF, TGF‐β_1_ and HIF‐1α antibodies were diluted with PBS buffer, placed in a humid box and incubated overnight at 4°C, with PBS buffer lotion; IgG antibody was added, and the solution was incubated at room temperature for 50 min, PBS buffer lotion was used, and a diaminobenzidine (DAB) was dropped. The colour development was stopped using distilled water. The sections were counterstained with haematoxylin for approximately 3 min, then with distilled water after the ammonia water turned blue and cover with a mounting medium. These were observed and photographed using the microscope and analysed.

### 2.7 Data analysis

SPSS software (version 21.0; Chicago, USA) was used for a one‐way ANOVA analysis of variance. The results were plotted with Grandpa Prism software (version 8.0; Chicago, USA) and expression levels as mean ± standard deviation (SD) in the histogram. *p* < 0.05 and 0.01 show significant and extremely significant differences, respectively.

## 3 RESULTS

### 3.1 mRNA expression of *VEGF*, *TGF‐β_1_* and *HIF‐1α* in follicles of different sizes

**FIGURE 1:**
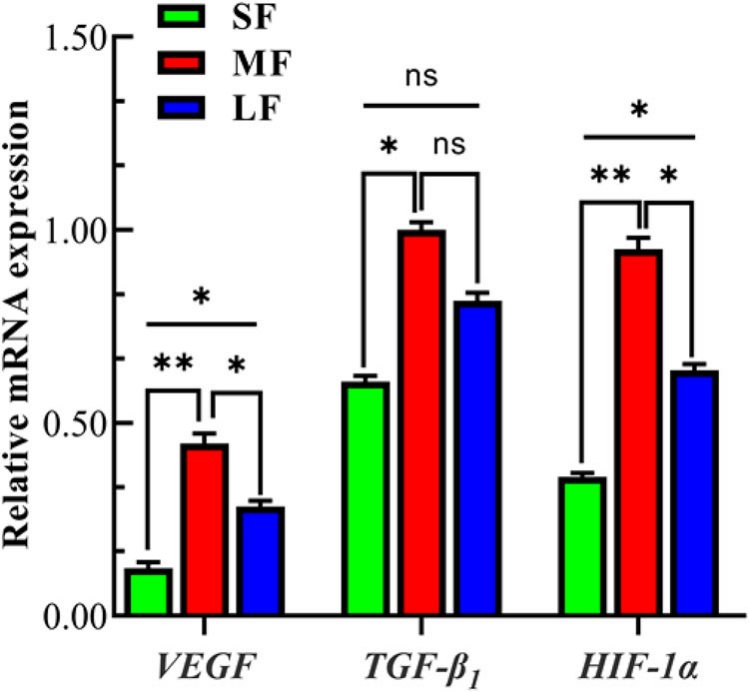
Relative abundance of *VEGF*, *TGF‐β_1_* and *HIF‐1α* transcription in different follicles of Tibetan sheep. *Note*: The results indicate the means ± SD. *β‐actin* was used as a reference gene. *p < 0.05; ns (no significance): *p* > 0.05. SF: small‐sized follicle; MF: medium‐sized follicle; LF: large‐sized follicle. The data are representative of at least three independent experiments

The relative expression levels of *VEGF*, *TGF‐β_1_* and *HIF‐1α* in different follicles were detected using qRT‐PCR, and it was found that the relative expression levels of *VEGF* in medium‐sized follicles were significantly higher than those in large‐ and small‐sized follicles (*p* < 0.05). There was no significant difference in the expression level of *VEGF* between small‐ and large‐sized follicles (*p >* 0.05); the expression level of *TGF‐β_1_* in the medium‐sized follicle was significantly higher than that in the small‐ and large‐sized follicles (*p* < 0.05); and the expression level of *HIF‐1α* in the medium‐sized follicle was significantly higher than that of the small‐ and large‐sized follicles (*p* < 0.05) (Figure 1). These results suggested that the transcriptional expression of these genes plays an important role in follicular development, especially in medium follicles.

### 3.2 Protein expression of VEGF, TGF‐β_1_ and HIF‐1α in different‐sized follicles

**FIGURE 2:**
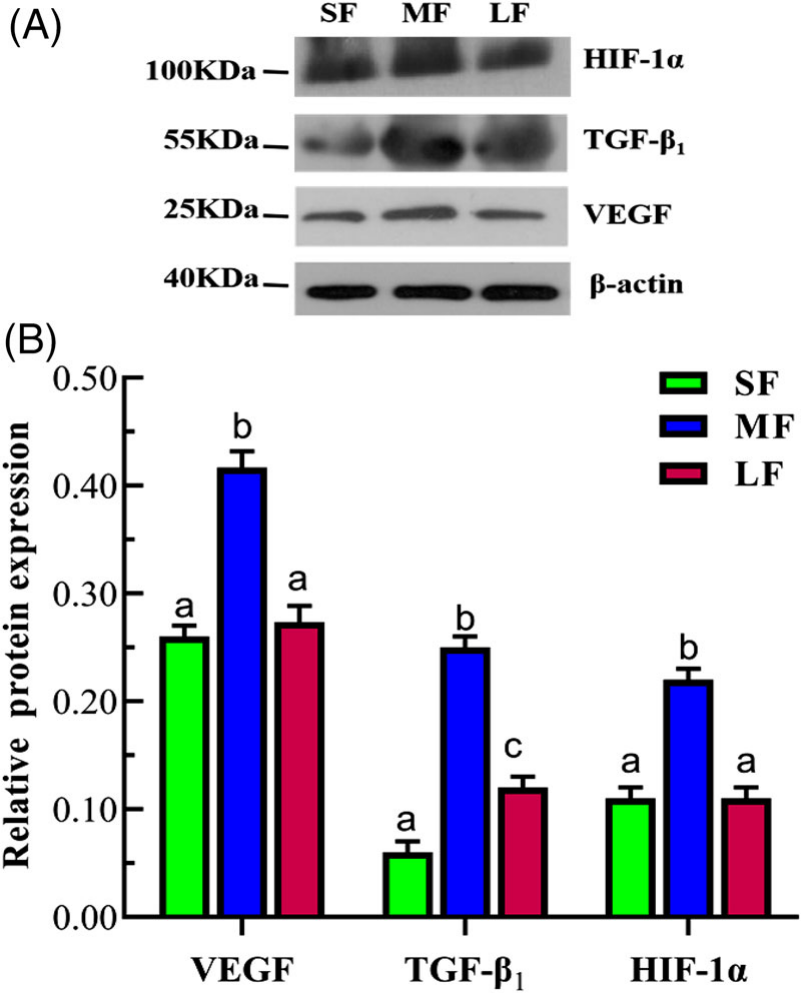
Relative abundance of VEGF, TGF‐β_1_ and HIF‐1α proteins in follicles. *Note*: The results have represented the means ± SD. β‐actin was used as a loading control. The same lowercase means no significant difference (*p* > 0.05); different lowercase means significant difference (*p* < 0.05). SF: small‐sized follicle; MF: medium‐sized follicle; LF: large‐sized follicle

Western blotting was used to detect and analyse the characteristics of VEGF, TGF‐β_1_ and HIF‐1α protein expression (Figure 2a). The results showed that the trend of their protein expressions was the same as those of the mRNA, the expression of VEGF, TGF‐β_1_ and HIF‐1α proteins in medium‐sized follicles were significantly higher than that in small‐ and large‐sized follicles (*p* < 0.05). Although the expression of VEGF and HIF‐1α in small‐ and large‐sized follicles did not differ significantly (*p* > 0.05), that of TGF‐β_1_ in large‐sized follicles was significantly higher than that in small‐sized follicles (*p* < 0.05) (Figure 2b). The outcome showed that these proteins were most significantly expressed in medium follicles, suggesting that they may play an important regulatory function during follicular development.

### 3.3 Immunolocalisation of VEGF, TGF‐β_1_ and HIF‐1α proteins in follicles of different sizes

**FIGURE 3:**
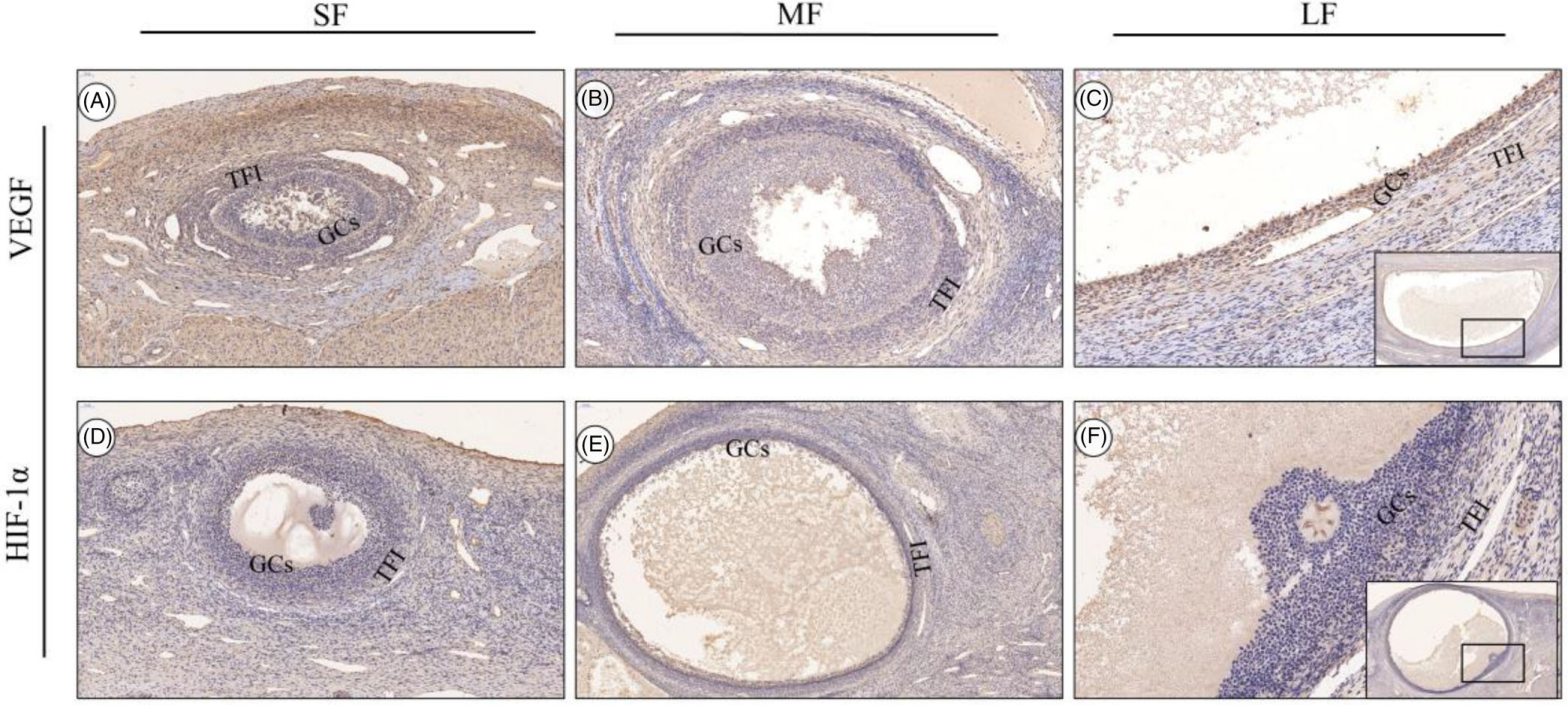
Immunostaining of VEGF and HIF‐1α protein in follicles of different sizes. (a, d) VEGF and HIF‐1α immunostaining in small‐sized follicles. (b, e) VEGF and HIF‐1α immunostaining in medium‐sized follicles. (c, f) VEGF and HIF‐1α immunostaining in large‐sized follicle. GCs: granulosa cells; TFI: theca folliculi interna

**FIGURE 4:**
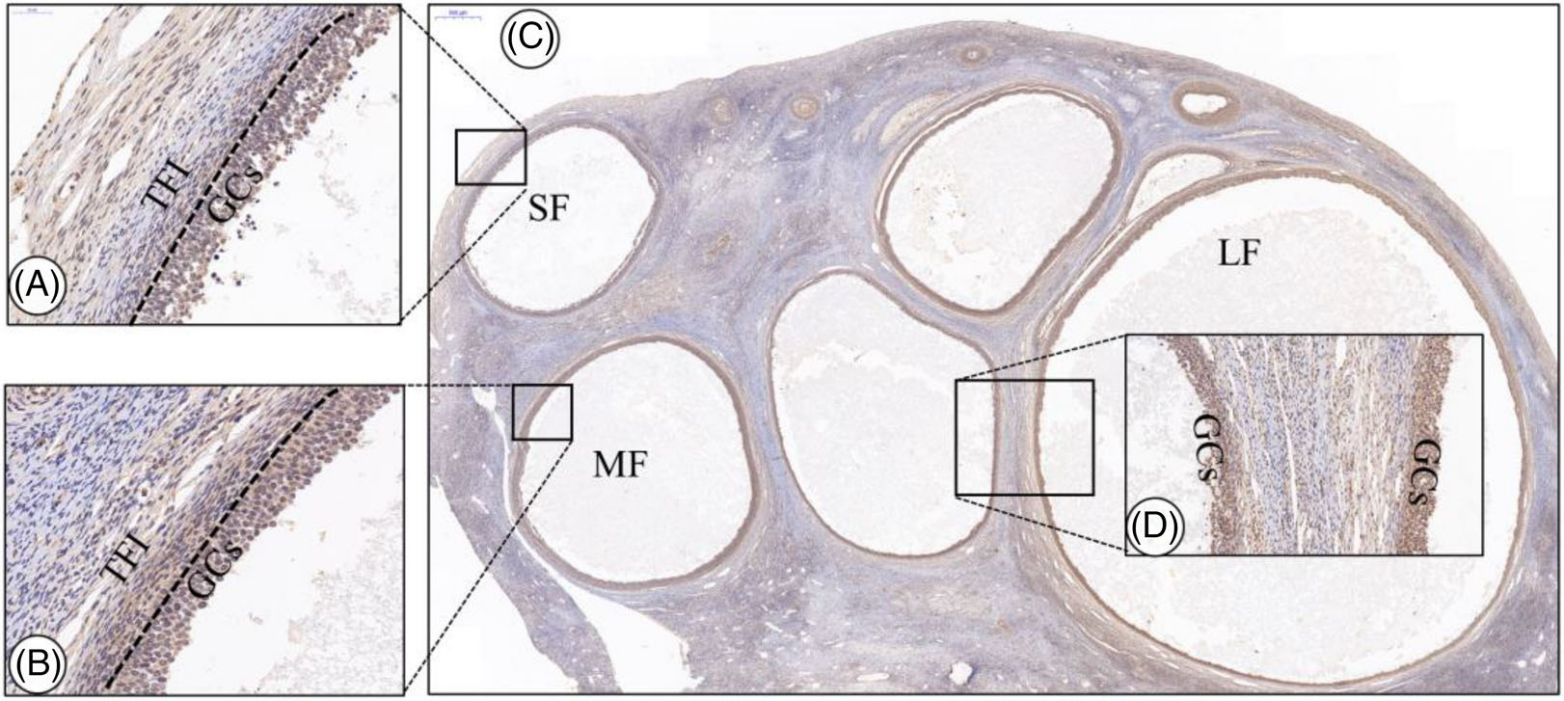
Immunostaining of TGF‐β_1_ protein in the follicles of different sizes. (a) TGF‐β_1_ immunostaining in small‐sized follicles. (b) TGF‐β_1_ immunostaining in medium‐sized follicles. (c) TGF‐β_1_ protein in the follicles of different sizes. (d) TGF‐β_1_ immunostaining in large‐sized follicle. GCs: granulosa cells; TFI: theca folliculi interna

IHC staining revealed that brown colour was the positive expression signal for VEGF, TGF‐β_1_ and HIF‐1α proteins and indicated the distribution and location of the three proteins in the corresponding follicles. The results showed that VEGF, TGF‐β_1_ and HIF‐1α proteins were positively expressed at different stages in the follicles of Tibetan sheep (Figures 3 and 4). VEGF, TGF‐β_1_ and HIF‐1α proteins showed positive signals in the follicular theca and GCs layers in different‐sized follicles. VEGF, TGF‐β_1_ and HIF‐1α were also present in theca folliculi interna (TFI). The positive signal strength of VEGF, HIF‐1α and TGF‐β_1_ in the GCs demonstrated their expression in medium‐, small‐ and large‐sized follicles (Figures 3a–f and 4a–d). The findings indicated a consistent spatiotemporal distribution of these proteins in GCs and TFI, which may be closely related to dominant follicle growth and steroid hormone secretion.

## 4 DISCUSSION

Our findings showed that the transcription and protein levels of VEGF, TGF‐β_1_ and HIF‐1α were shown to be coincident in follicles. One study found that HIF‐1α stimulates the expression of VEGF during the formation of mammalian follicles, according to earlier studies (Alam et al., 2009; Cockman et al., 2000). VEGF has been linked to follicular development, optimisation, maturity and ovulation (Cockman et al., 2000; Geva & Jaffe, 2000; Kawano et al., 2003; Stouffer et al., 2001). VEGF and HIF‐1α have also been determined to have a strong relation in studies (Alam et al., 2009; Cockman et al., 2000). These studies showed that VEGF expression is associated with folliculogenesis. Moreover, HIF‐1α is hypothesised to activate the downstream processes of steroid biosynthesis and GCs proliferation in the gonadotropin response, many of which are critical for ovulation (Baddela et al., 2020; Fadhillah et al., 2017). FSH increases the size of follicles while inhibiting VEGF expression, thus according to studies, and can prevent the formation of antral follicles, steroid secretion and membrane vascularisation (Kowalewski et al., 2015; Tam et al., 2010). These findings suggest that they may co‐participate in follicular growth and development, performing a significant role in follicular development. Furthermore, the TGF‐β_1_ appeared to have similar effects (Juengel & McNatty, 2005). TGF‐β_1_ may stimulate FSH induced aromatase activity, inhibin production, luteinising hormone (LH) production and luteinising hormonereceptor (LHR) induction via stimulating FSH receptor expression (Demeter‐Arlotto et al., 1993; Drummond et al., 2000; Hutchinson et al., 1987). The SMAD2/3, ERK1/2 and p38 MAPK signalling pathways have been implicated in the synthesis and secretion of VEGF stimulated by TGF‐β_1_, according to research (Fang et al., 2020). Our findings demonstrated that TGF‐β_1_ and VEGF expression patterns and features in follicles were identical, which is consistent with the research (Fang et al., 2020). TGF‐β_1_ and VEGF transcription levels in Tibetan sheep follicles are positively associated, demonstrating that VEGF participates in the TGF‐β_1_ regulatory pathway and then influences follicular development and GCs proliferation. The proteins VEGF, TGF‐β_1_ and HIF‐1α in the theca cell and GCs of different follicles appeared to still be positive signals in this investigation. There are related studies with similar results, such as those on humans (*Homo sapiens*) (Neulen et al., 1998), pigs (*Sus scrofa*) (Barboni et al., 2000; Shimizu et al., 2002), rats (*Rattus norvegicus*) (Carmelite et al., 1996), cattle (*Bos taurus*) (Berisha et al., 2000; Greenaway et al., 2004), sheep (*Ovis aries*) (Chowdhury et al., 2010) and buffaloes (*Bubalus bubalis*) (Babitha et al., 2013), while VEGF expression can be detected with an increase in the expression level as the follicular growth. Other research showed that the development of the vascular network in the follicles was closely related to the expression of VEGF in GCs and the expression of Flt‐1 and KDR in follicular theca cells (Shimizu et al., 2002; Wang et al., 2020). Many animal models, including mice (Kim et al., 2009), cattle (Berisha et al., 2017), pigs (Boonyaprakob et al., 2005) and humans (Herr et al., 2004; Henrquez et al., 2017), have been reported to possess the HIF‐1α protein in their GCs. HIF‐1α is also expressed in mature mouse oocytes and continues to be expressed after fertilisation (Takahashi et al., 2016). Nevertheless, this finding differed from previous findings. The study demonstrated that the mouse stomach antral follicles have minimal levels of HIF‐1α activity (Kind et al., 2015), suggesting that there is no hypoxia during follicle growth to support follicle metabolic demands. In addition, TGF‐β_1_, which is generated by the theca cell and GCs of the ovarian follicle, was been recognised in ovarian cells (Juengel & McNatty, 2005). There was consistent with our results, showing the expression profiles of TGF‐β_1_ in follicles at different sizes, as well as their localisation in the GCs and TFI. These date indicate that VEGF, TGF‐β_1_, and HIF‐1α may play an important regulatory role in follicular growth and development. However, further research is required into the specific regulatory mechanisms of VEGF, TGF‐β_1_ and HIF‐1α in the growth and maturation of follicles, as well as the proliferation and differentiation of GCs.

## 5 CONCLUSION

In summary, this study provides the first demonstration of the expression of VEGF, TGF‐β_1_ and HIF‐1α in medium‐, large‐ and small‐sized follicles as well as GCs were distributed. Importantly, it indicates that the progressive growth of follicles is under the control of VEGF, TGF‐β_1_ and HIF‐1α that act in an autocrine or paracrine fashion within GCs, suggesting these growth factors are associated with follicular growth and development.

## AUTHOR CONTRIBUTIONS

Yajun Guo: Formal analysis, investigation, methodology and writing‐original draft. Miaomiao Liu and Jos Dorian Lawson Mfoundou: Conceptualisation, data curation, supervision, validation and visualisation. Yajun Guo and Xinrong Wang: Methodology, resources, supervision, validation, visualisation, writing‐review and editing.

## CONFLICT OF INTEREST

The authors have no conflict of interest to declare.

### PEER REVIEW

The peer review history for this article is available at https://publons.com/publon/10.1002/vms3.907

## ETHICS STATEMENT

All animal experiments were conducted according to the guidelines for the care and use of experimental animals established by the Ministry of Science and Technology of the People's Republic of China (approval number: 2006–398), and the work was approved by the Experimental Animal Care and Use Committee of the Gansu Agricultural University (GAU‐LL‐2020‐48), Lanzhou, China.

## Data Availability

The data that support the findings of this study are available from the corresponding author upon reasonable request.
